# Risk Factors and Medico-Economic Effect of Pancreatic Fistula after Pancreaticoduodenectomy

**DOI:** 10.1155/2015/917689

**Published:** 2015-02-19

**Authors:** Renping Huang, Bing Liu, Hua Chen, Xuewei Bai, Rui Kong, Gang Wang, Yongwei Wang, Bei Sun, Yinghui Guan

**Affiliations:** ^1^Vascular Surgery, Department of General Surgery, The First Affiliated Hospital of Harbin Medical University, Harbin 150001, China; ^2^Department of Pancreatic and Biliary Surgery, The First Affiliated Hospital of Harbin Medical University, Harbin 150001, China

## Abstract

The study aimed to uncover the risk factors for the new defined pancreatic fistula (PF) and clinical related PF (CR-PF) after pancreaticoduodenectomy (PD) surgery and to evaluate the medico-economic effect of patients. A total of 412 patients were classified into two groups according to different criteria, PF and NOPF according to PF occurrence: CR-PF (grades B and C) and NOCR-PF (grade A) based on PF severity. A total of 28 factors were evaluated by univariate and multivariate logistic regression test. Hospital charges and stays of these patients were assessed. The results showed that more hospital stages and charges are needed for patients in PF and CR-PF groups than in NOPF and NOCR-PF groups (*P* < 0.05). The excessive drinking, soft remnant pancreas, preoperative albumin, and intraoperative blood transfusion are risk factors affecting both PF and CR-PF incidence. More professional surgeons can effectively reduce the PF and CR-PF incidence. Patients with PF and CR-PF need more hospital costs and stages than that in NOPF and NOCR-PF groups. It is critical that surgeons know the risk factors related to PF and CR-PF so as to take corresponding therapeutic regimens for each patient.

## 1. Introduction

Pancreaticoduodenectomy (PD) is performed for treatment of patients with benign and malignant pancreatic and periampullary diseases. Despite improved surgical technique and postoperative care, the mortality of PD is high with mortality rate up to 30%, due to its complex and challenging surgical procedure and high incidence of postoperative complications [1–4].

Pancreatic fistula (PF) is the one of the most frequent complications of PD and occurs when pancreatic anastomosis fails to heal during surgery [5, 6]. A definition and clinical classification of PF were proposed by the international study group of PF (ISGPF) in July, 2005 [1]. PF is defined as either a measurable drainage from an operatively place or a continuous placed percutaneous drain with amylase at least 3× normal serum activity 3 days postoperatively. The ISGPF classified the PF severity into grades A, B, and C based on the symptoms and treatment demand: grade A of PF is transient, asymptomatic fistulas, just with elevated drain amylase levels; grade B is clinical apparent fistulas that need diagnostic assessment and therapeutic management; grade C is severe and requires major deviations in clinical management. Patient, who is diagnosed as grades B and C, develops a clinical relevant PF (CR-PF).

The treatment of PF with an incidence ranging from 9.9% to 28.5% [7] will no doubt prolong the postoperative recovery time and hospital stays and elevate the hospital cost and mortality of PD patients. Recent literatures have suggested that many factors could influence PF after PD, such as age, sex, operative time, anastomotic technique, intraoperative blood loss, remnant pancreas texture, use of somatostatin, jaundice, and surgeons experience [3, 8–12]. However, the research about if these risk-related factors have impact on the new defined and classified PF was relatively deficient. To evaluate the potential risk factors for PF and CR-PF patients after PD and to further access the medico-economic consequences of these patients, we collected data of 412 patients who underwent PD during January 2007 and June 2014 and analyzed by the univariate and multivariate tests in the present study.

## 2. Materials and Methods

### 2.1. Patient Selection and Characteristics

Data of consecutive patients who underwent PD surgery at our hospital between January 2007 and June 2014 was collected in our study. Patients were excluded if (a) they had incomplete information; (b) they performed entire pancreatectomy; (c) they died during the PD operation or after operation within 3 days. According to these exclusion criteria, 34 patients were excluded.

Medical records of included patients were entered into a database, including gender, ages, body mass index (BMI), smoking status, alcohol drinking status (excessive drinking or not: excessive drinking is defined blood alcoholicity of more than 0.08), preoperative complications (such as coronary heart disease: this disease was determined by confirmed history of myocardial infarction, angina, or coronary revascularization), pathological diagnosis, diseased region, operative duration, amount of intraoperative bleeding, amount of intraoperative blood transfusion, residual pancreatic texture, pancreatic duct diameter, biochemical index in pre- and postoperation, volume of abdominal drainage, amylase content in abdominal drainage, postoperative regimen, and hospital stays and hospital charges. These patients with the occurrence of PF were grouped into PF. The rest of PD patients without PF the occurrence of were defined as NOPF and grouped into NOPF. CR-PF was defined as PF patients diagnosed as grade B fistulas and grade C fistulas that needed clinical intervention, and NOCR-PF were defined as non-PF patients and grade A PF patients that did not need clinical intervention.

### 2.2. Surgical Methods and Postoperative Care

PD was performed with or without pylorus-preservation (PP) by either laparoscopic operation or laparotomy. The reconstruction of digestive tract was conducted by anastomosis including binding anastomosis to the jejunum, end-end invagination anastomosis, end-side invagination anastomosis, and duct-mucosa anastomosis. Pancreatic duct stent was applied in some patients. One or two drainage tubes were placed at the anastomotic stoma of all surgeries.

Surgeons who performed PD operations ≥ 10 times per year were considered as professional, the others were considered as nonprofessional. Among all PD operations of our patients, 228 were performed by the professional surgeons and 184 were performed by unprofessional surgeons. Patients who had more than 300 *μ*mol/L total bilirubin underwent biliary drainage during the operation. Those whose serum albumin was less than 30 g/L in perioperative period were supplemented with albumin. Patients with hemoglobin less than 70 g/L in perioperative period were treated with transfusion. Some patients were treated with somatostatin after surgery.

### 2.3. Statistical Analysis

Statistical analysis was performed by SPSS version 18.0 software. Data were expressed as *x* ± *s*. Categorical variables were analyzed by Fisher's exact test and chi-square test, and comparison of quantitative variables was analyzed by independent sample *t*-test. Variables reaching a *P* value of < 0.05 in a univariate analysis were included in the multivariate analysis by using a logistic regression model. The results of logistic regression model were assessed for independence of risk factors. Statistical significance was defined at the *P* < 0.05 level.

## 3. Results

### 3.1. Demographic Characteristics of PD Patients

A total of 412 patients (260 men and 152 women) who underwent PD, with a mean age of 56 years (range from 22 to 79) undergoing PD between January 2007 and June 2014 were enrolled in our study. PF occurred in 126 (30.58%) of them, who were grouped into PF. The other 286 (69.42%) patients without the occurrence of PF were grouped into NOPF. Details regarding the characteristics of these patients were listed in Table 1. Among PF patients, 52 were diagnosed as A grade, 58 were diagnosed as B grade, and 16 were diagnosed as C grade. There were no significant statistical differences in genders, age groups (<70 and ≥70), and BMI between PF and NOPF groups, as well as CR-PF and NOCR-PF groups (Table 1). Unfortunately, 9 of them (2.18%) died after surgery.

### 3.2. Potential-Related Factors for PF

Univariate logistic regression analysis showed that (Table 2(a)) there were significant associations between PF occurrence rates and patient-related factors of excessive drinking (*P* = 0.029), coronary heart disease (*P* = 0.029), and preoperative albumin (*P* = 0.006). Among comorbidities, a history of cholangitis, cholecystitis, jaundice, hypertension, or diabetes mellitus was similar in PF and NOPF groups. Besides, the smoking habits, preoperative serum bilirubin, postoperative serum albumin, primary site of disease, pathologic diagnosis, and pancreatic duct diameter were also found to have no association with PF incidence.

Univariate logistic regression analysis of operative- and therapeutic-related factors in PF and NOPF groups was showed in Table 2(b). There was no significant difference in PF rates between the preoperative biliary drainage treatment or not. Early jejunal nutrition, operative time, and use of somatostatin after PD were also found to have no correlation with PF rates. On the contrary, the pancreatic duct stent drainage methods, excision methods, anastomosis methods, intraoperative blood loss (*P* = 0.003), intraoperative blood transfusion (*P* = 0.000), pancreatic duct stent drainage (*P* = 0.000), excision methods (*P* = 0.016), methods of anastomosis (*P* = 0.005), intraoperative blood transfusion (*P* = 0.000), laparoscopic operation or not (*P* = 0.002), and professional group or not (*P* = 0.000) markedly influenced the rate of PF.

### 3.3. Independence Risk Factors for PF

Based on the results of the above univariate analysis, additional multivariate analysis was performed for evaluating the independence of risk factors. As is showed in Table 3, both the excessive drinking and coronary heart disease were proved to be the independent risk factors for PF with odds ratio (ORs) of 0.390 (95% CI = (0.210–0.724), *P* = 0.003) and 0.324 (95% CI = (0.127–0.828), *P* = 0.018), respectively. The preoperative albumin (*P* = 0.007) was significantly higher in the PF group than in the NOPF group (Table 3(a)). More intraoperative blood transfusion (*P* = 0.000) and harder remnant pancreas texture (*P* = 0.037) significantly reduced the PF risk. In addition, different methods of anastomosis, laparoscopic operation, and professional group were also included in the independent risk factors affecting PF (*P* = 0.026). Though pancreatic duct stent drainage and excision method were proved to be associated with PF in univariate analysis, the multivariate analysis showed that they were not independent risk factors (*P* > 0.05) (Table 3(b)).

### 3.4. Potential-Related Factors for CR-PF

Univariate logistic regression analysis showed that (Table 3(a)) the patient characteristics such as cholangitis (*P* = 0.002), preoperative serum albumin (*P* = 0.000), and texture of the remnant pancreas (*P* = 0.013) were significantly related to the CR-PF. The other patient characteristics, for example, smoking, excessive drinking, cholecystitis, jaundice, coronary heart disease, hypertension, diabetes mellitus, preoperative serum total bilirubin, preoperative hemoglobin, postoperative serum albumin, primary site of disease, pathologic diagnosis, and diameter of pancreatic duct, had no influence on CR-PF occurrence (all *P* > 0.05).

The operative and therapeutic risk factors such as intraoperative blood loss (*P* = 0.004), intraoperative blood transfusion (*P* = 0.002), pancreatic duct stent drainage (*P* = 0.007), and professional group were associated with an increased incidence of CR-PF (*P* = 0.000), while the left factors were found to have no significant association with the risk of CR-PF (Table 3(b)).

### 3.5. Independence Risk Factors for ORPF

When assessing the independent effect of the potential risk factors on ORPF occurrence in multivariate analysis, cholangitis, preoperative albumin, intraoperative blood transfusion, texture of the remnant pancreas, and professional group or not were the significant associated factors (all *P* < 0.05), whereas the effect of pancreatic duct stent drainage methods had no independent effect on ORPF.

### 3.6. Hospital Charges and Hospital Stays

Mean hospital stays were shorter in the NOPF group and NOCR-PF patients than in the PF and CR-PF patients, respectively (Table 4). By use of nonparametric test analysis, there were significant differences in hospital stays between PF and NOPF, as well as between CR-PF and NOCR-PF (both *P* values were 0.000). The mean charges of the PF and NOPF patients were 56323.47 RMB and 83347.93 RMB, respectively, which exhibited significant difference with each other by *t* text (*P* = 0.000). Similarly, significant difference was found between CR-PF and NOCR-PF groups as well (*P* = 0.001), with mean hospital charges of RMB 61339.84 and 81448.18, respectively.

## 4. Discussion

Effective management of PF has proven to be one of the most intractable challenges after PD surgery. Confront with this adversity, there has been a shift therapeutic regimen for management of PF, from a reactive “wait and see” to a proactive strategy that relies on early anticipation and timely prevention [9, 13]. However, this approach depended on assumption and prediction of the risk for PF development. In the present study, we collected clinical data of 412 patients in our hospital, analyzed the potential risk factors associated with PF and CR-PF, and evaluated the medico-economic effect on these patients. Our results showed that the excessive drinking, coronary heart disease, preoperative albumin, intraoperative blood transfusion, texture of the remnant pancreas, methods of anastomosis, laparoscopic operation, and professional group were independently associated with PF occurrence. Among these risk factors of PF, the preoperative albumin, intraoperative blood transfusion, texture of the remnant pancreas, and professional group were significantly and independently associated with CR-PF. Though history of cholangitis in patients was found insignificantly related with PF, it was one of the risk factors that affected CR-PF significantly.

Although risk factors for PF and CR-PF have historically been reported in the literature, their relevance in application has been hampered by definitions of fistula [14]. Actually, several studies have tried to identify the risk factors associated with the PF development and many risk factors have been proposed. However, only a few factors are independent factors of PF and they vary among different studies. In the present study, total occurrence rate of PF after PD surgery was 30.58%, which was slightly higher than previous results. The reason may be explained by the different definitions of PF. Though a normalized definition was proposed by ISGPF, the definition of PF might in some case not be specific, because it includes asymptomatic patients who are not clinically ill [6]. Therefore, the study of risk factors affecting grades B and C will be more meaningful in clinical practice.

Generally, PF risk evaluation begins in the preoperative setting, such as patient-related factors. In this study, we retrospectively analyzed the conditions of PF patients before PD surgery and found that the preoperative serum albumin, history of coronary heart disease, and excessive drinking were the independent risk factors associated with PF. With respect to CR-PF, the independent risk factors were preoperative serum albumin and cholangitis.

Albumin in serum has properties of maintaining normal plasma osmotic pressure [15] and acid-base balance [16], antioxidant [17], scavenging free radical [18], and protecting microcirculation [19]. On the one hand, tissue edema caused by hypoproteinemia may lead to undesirable or anastomotic stoma healing and then increase the incidence of PF or CR-PF. On the other hand, the increased hypoproteinemia complications such as infection and diarrhea will influence the PF and CR-PF more or less.

There is evidence from the current literatures that cardiovascular disease is a risk factor for PF [20], which was consistent with our findings, while the effect of coronary heart disease on CR-PF was not significant. The reasons why coronary heart disease would be associated with PF are not well understood. Perhaps the cardiovascular and cerebrovascular diseases are surrogate for decreased visceral perfusion result in anastomotic ischemia, or perhaps the related medications to such patients compromise anastomotic healing [21]. Therefore, the association between cardiovascular disease and PF should be well explained by reliable evidence from clinical outcomes.

Animal experiments and epidemiological studies have suggested that alcohol had toxicity to pancreas [22, 23]. It was proved to be an independent risk factor for PF occurrence in our patients undergoing PD. There are several hypotheses on the toxicity mechanisms of ethanol to pancreas. (a) The ethanol has toxic effect on pancreatic acinar cells and disturbs its metabolism [24]. (b) The accumulation of pancreatic stone protein induced by ethanol produces ulceration and inflammation of the ductules, and the ductule then leads to atrophy, insufficiency, and fibrosis themselves [25]. (c) The disorders such as sphincter of Oddi dysfunction caused by ethanol have a connection with stenosis of ductule and regurgitation of duodenal juice [25]. (d) Excessive drinking will destroy the essential minerals and induce the oxygen radical* in vivo* in human, which are harmful to pancreas [24]. We believe that the excessive drinking effect on PF will be closely related to its effect on pancreas.

One of the interesting findings in our research is that patients with cholangitis will be more likely to suffer from CR-PF than noncholangitis patients. Generally, mucosa in biliary ducts is congestive in cholangitis patients, especially in patients with obstruction of biliary tract. Edema and inflammation usually happen in pancreatic tissues when the bile duct enlarged by obstruction. Therefore, it is harmful to conduct the anastomosis and CR-PF is likely to occur. In addition, cholangitis is usually accompanied with increased bacteria in the bile duct. The increased intraductal pressure can lead to bacteria translocation or endotoxemia in these patients [26], which may be another indirect factors affecting CR-PF occurrence.

The factors of pathologic diagnosis, texture of the remnant pancreas and diameter of pancreatic duct have been widely accepted as the related risk factors of PF [12, 27–29]. Logistic analysis of regression showed that patients with soft texture of the remnant pancreas had higher PF and CR-PF incidence than that in patients with hard texture. There are several explanations for this association. Firstly, a soft pancreas is more susceptible to injury and ischemia during operative dissection [29]. Meanwhile, exocrine function is usually preserved in the soft pancreas, leading to increased secretion of pancreatic juice and activation of proteolytic enzymes, which is more prone for PF development [30]. However, our data did not provide evidence to support pathologic diagnosis and diameter of pancreatic duct (≥3 mm and <3 mm) of influence factors for PF and CR-PF. The relationship between them needs more studies in the future.

Blood transfusion in response to blood loss is considered to be an immunosuppressive effect. In our study, the intraoperative blood transfusion was mainly caused by intraoperative blood loss, preoperative anemia, coagulation disorders, and so forth. Though our results showed that the intraoperative blood transfusion was the risk factor influencing both PF and CR-PF, the full impact of intraoperative blood transfusion is not well understood. Rapid blood loss, as well as anemia and coagulation disorder, may cause ischemia and poor healing of the pancreatic anastomosis, because of tissue edema from aggressive blood replacement in a “rebound” fashion [29]. In addition, other adverse effects such as complications of blood transfusion, disseminated intravascular coagulation, and hemorrhagic tendency during intraoperative blood transfusion may increase the risk of PF and CR-PF after PD surgery.

Hypertension has been noted as one of the risk factors in previous studies [31, 32]. They assumed that the pathophysiological effects of hypertension caused generalized atherosclerosis and therefore limited the microcirculation of the tissue. That will negatively affect the healing process of PD. However, in our study, we found no significant association between hypertension and postoperative PD. Therefore, we strongly recommended more studies to resolve these controversial results.

The PD surgery with laparoscope has been clinically applied since its first description by Gagner and Pomp [33] in 1994 [34, 35]. The multivariate analysis of 13 PD patients with laparoscope (PF: 10, CR-PF: 4) and 309 patients with laparotomy showed that more patients treated with laparoscope developed PF than that treated with laparotomy. Fortunately, the laparoscope treatment had no significant side effect on CR-PF. Limitations of this approach including inability to palpate the lesion, relatively narrow view, inaccurate location, and misestimates of tumor spread may be responsible for high PF occurrence. We believe that these limitations will be minimized as the experiential accumulation and technological improvement.

Other operative- and therapeutic-related factors such as treatment of residual pancreatic, application of pancreatic duct stent drainage, methods of anastomose, use of somatostatin after PD, and excision methods have suggested associating with the PF incidence [21, 32, 36]. Analysis of these factors in our study revealed that just anastomose methods were associated with PF. The binding anastomosis was proved to be superior to end-side invagination anastomosis, end-end invagination anastomosis, and duct-mucosa anastomosis, because of its less PF incidence. Instead of suture, binding anastomosis can definitely minimize the leakage by avoiding any pinhole through the closure [37]. Furthermore, binding anastomosis avoids the regurgitation of pancreatic juice by maintaining higher blasting pressure in jejunum than other methods [38]. However, this method was found to have no relationship with CR-PF.

Unquestionably, the complex and difficult PD operation is a challenge to surgeons. Therefore the skilled and experienced surgeons will be important factors related to PF incidence. In our study, the PF and CR-PF incidence after PD surgery in professional group were 3 and 5.7 times more than nonprofessional group, respectively. The high-volume surgeons were proved to have lower PF rate [39, 40], probably due to more experience for surgeons. Therefore, it is necessary for training PD surgeons and establishing professional group in future.

Patients with PF, especially with CR-PF after PD surgery, usually have to prolong the hospital stages and pay more for external surgery than patients in NOPF or NOCR-PF group. Our study also showed more hospital stages and charges in PF and CR-PF groups than in NOPF and NOCR-PF groups. Future studies that address the charge and hospital stages are required, in light of the rapid increase of technology.

## 5. Conclusion

In summary, the excessive drinking, coronary heart disease, preoperative albumin, intraoperative blood transfusion (>600 mL), soft remnant pancreas, and laparoscopic operation were risk factors affecting PF incidence after PD. Binding anastomosis between remnant pancreas and jejunum can effectively reduce the PF incidence compared with the other anastomosis methods, such as end-side invagination anastomosis, end-end invagination anastomosis, and duct-mucosa anastomosis. The risk factors such as cholangitis, hypoproteinemia, intraoperative blood transfusion volume (>300 mL), and soft remnant pancreas were significantly associated with CR-PF. Surgeons with more experience and profession can significantly reduce the PF and CR-PF incidence when they perform PD surgery. More hospital stages and charges in PF and CR-PF groups are needed than in NOPF and NOCR-PF groups. It is critical that surgeons know the risk factors related to PF and CR-PF so as to take corresponding therapeutic regimens for each patient.

## Figures and Tables

**Table 1:** Demographic characteristics description.

Variants	PF	NOPF	Total	*χ* ^2^ (*P* value)	CR-PF	NOCR-PF	Total	*χ* ^2^ (*P* value)
	No. (%)	No. (%)	No. (%)		No. (%)	No. (%)	No. (%)	
Gender				0.597 (0.440)				0.515 (0.473)
Male	83 (65.9)	177 (61.9)	260 (63.1)		44 (59.5)	216 (63.9)	260 (63.1)	
Female	43 (34.1)	109 (38.1)	152 (36.9)		30 (40.5)	122 (36.1)	152 (36.9)	
Age (year)				0.077 (0.782)				0.619 (0.431)
<70	113 (89.7)	259 (90.6)	372 (90.3)		65 (87.8)	307 (90.8)	372 (90.3)	
≥70	13 (10.3)	27 (9.4)	40 (9.7)		9 (12.2)	31 (9.2)	40 (9.7)	
BMI				1.951 (0.377)				0.328 (0.849)
<18.5	14 (11.1)	41 (14.3)	55 (13.3)		9 (12.2)	46 (13.6)	55 (13.3)	
18.5–25	90 (71.4)	208 (72.7)	298 (72.3)		53 (71.6)	245 (72.5)	298 (72.3)	
≥25	22 (17.5)	37 (12.9)	59 (14.3)		12 (16.2)	47 (13.9)	59 (14.3)	

PF: patients undergoing pancreatic fistula after pancreaticoduodenectomy (PD); NOPF: PD patients without PF occur; CR-PF: PF patients diagnosed as grade B fistulas and grade C fistulas; NOCR-PF: non-PF patients and grade A PF patients; BMI: body mass index.

**Table:** (a) Patient-related factors for PF

Variants	PF	NOPF	Univariate analysis		Multivariate analysis	
	No. (%)	No. (%)	OR_adj_ (95% CI)	*P*	OR_adj_ (95% CI)	*P*
Smoking				0.499		
Yes	34 (27.0)	68 (23.8)	1			
No	92 (73.0)	218 (76.2)	0.847 (0.524–1.370)			
Heavy smoking (cigarettes/day)				0.17		
≥20	24 (19.0)	39 (13.6)	1			
<20	102 (81.0)	247 (86.4)	0.675 (0.385–1.184)			
Excessive drinking				0.029		0.003
Yes	35 (27.8)	53 (18.5)	1		1	
No	91 (72.2)	233 (81.5)	0.577 (0.352–0.947)		0.390 (0.210–0.724)	
Cholangitis				0.06		
Yes	21 (16.7)	29 (10.1)	1			
No	105 (83.3)	257 (89.9)	0.577 (0.303–1.024)			
Cholecystitis				0.054		
Yes	105 (83.3)	213 (74.5)	1			
No	21 (16.7)	73 (25.5)	0.588 (0.342–1.010)			
Jaundice				0.671		
Yes	87 (69.0)	202 (70.6)	1			
No	39 (31.0)	84 (29.4)	1.104 (0.698–1.747)			
Coronary heart disease				0.029		0.018
Yes	16 (12.7)	16 (5.6)	1		1	
No	110 (87.3)	270 (94.4)	0.441 (0.211–0.919)		0.324 (0.127–0.828)	
Hypertension				0.353		
Yes	17 (13.6)	26 (9.1)	1			
No	108 (86.4)	259 (90.9)	0.728 (0.373–1.422)			
Diabetes mellitus				0.624		
Yes	14 (11.2)	27 (9.5)	1			
No	111 (88.8)	257 (90.5)	0.842 (0.424–1.674)			
Preoperative serum total bilirubin (*μ*mol/L)				0.83		
≤17.1	28 (22.2)	63 (22.0)	1			
>17.1	98 (77.8)	223 (78.0)	0.946 (0.568–1.573)			
Preoperative hemoglobin (g/L)				0.964		
<90	3 (2.4)	7 (2.4)	1			
≥90	123 (97.6)	279 (97.6)	1.032 (0.261–4.087)			
Preoperative serum albumin (g/L)				0.006		
<30	10 (7.9)	6 (2.1)	1		1	0.007
≥30	116 (92.1)	280 (97.9)	0.235 (0.083–0.666)		0.182 (0.053–0.626)	
Postoperative serum albumin (g/L)				0.095		
<30	51 (40.5)	93 (32.5)	1			
≥30	75 (59.5)	193 (67.5)	0.689 (0.445–1.067)			
Primary site of disease				0.674		
Caput pancreatis	59 (46.8)	129 (45.1)	1			
Duodenum	34 (27.0)	89 (31.1)	0.815 (0.492–1.349)			
Biliary ducts	33 (26.2)	68 (23.8)	1.019 (0.605–1.717)			
Pathologic diagnosis				0.371		
Caput pancreatis cancer	48 (38.1)	91 (31.8)	1			
Duodenal cancer	21 (16.7)	60 (21.0)	0.642 (0.348–1.184)			
Cholangiocarcinoma	30 (23.8)	62 (21.7)	0.906 (0.516–1.590)			
Pancreatitis	4 (3.2)	21 (7.3)	0.416 (0.134–1.294)			
Carcinoma of ampulla	6 (4.8)	20 (7.0)	0.588 (0.220–1.570)			
Others	17 (13.5)	32 (11.2)	1.141 (0.568–2.295)			
Texture of the remnant pancreas				0.016		0.037
Hard	20 (15.9)	77 (26.9)	1		1	
Soft	106 (84.1)	209 (73.1)	1.964 (1.136–3.394)		1.955 (1.042–3.669)	
Diameter of pancreatic duct (mm)				0.496		
<3	68 (54.0)	142 (49.7)	1			
≥3	58 (46.0)	144 (50.3)	0.863 (0.566–1.318)			

**Table:** (b) Operative- and therapeutic-related factors for PF

Variants	PF	NOPF	Univariate analysis		Multivariate analysis	
	No. (%)	No. (%)	OR_adj_ (95% CI)	*P*	OR_adj_ (95% CI)	*P*
Preoperative biliary drainage treatment						
Yes	13 (10.3)	25 (8.7)	1	0.535		
No	113 (89.7)	261 (91.3)	0.798 (0.392–1.625)			
Operative time (min)						—
<295	19 (15.1)	67 (22.9)	1	0.161		—
≥295	107 (84.9)	226 (77.1)	0.669 (0.380–1.177)			—
Intraoperative blood loss (mL)				0.003	—	—
<300	35 (27.8)	121 (42.3)	1		—	—
300–600	30 (23.8)	81 (28.3)	1.194 (0.675–2.113)		—	—
600–900	28 (22.2)	45 (15.7)	2.089 (1.139–3.830)		—	—
≥900	33 (26.2)	39 (13.6)	2.738 (1.498–5.005)		—	—
Intraoperative blood transfusion (mL)				0.000		0.000
<300	41 (32.5)	136 (47.6)	1		1	
300–600	23 (18.3)	67 (23.4)	1.112 (0.616–2.008)		1.128 (0.556–2.290)	0.738
600–900	31 (24.6)	57 (19.9)	1.754 (1.000–3.078)		2.574 (1.318–5.025)	0.006
≥900	31 (24.6)	26 (9.1)	3.711 (1.969–6.995)		5.115 (2.364–11.069)	0.000
Pancreatic duct stent drainage				0.000		0.394
No stent	40 (31.7)	49 (17.1)	1		1	0.570
Internal drainage	77 (61.1)	174 (60.8)	0.518 (0.313–0.856)		0.819 (0.412–1.629)	0.178
External drainage	9 (7.1)	63 (22.0)	0.162 (0.071–0.370)		0.476 (0.161–1.403)	0.624
Excision method				0.016		0.624
Without PP	108 (85.7)	266 (93.0)	1		1	
With PP	18 (14.3)	20 (7.0)	2.300 (1.165–4.543)		1.240 (0.524–2.932)	
Methods of anastomosis				0.005		0.026
Binding anastomosis	3 (2.4)	22 (7.7)	1		1	
End-side invagination anastomosis	6 (4.8)	38 (13.3)	1.110 (0.251–4.911)		2.428 (0.439–13.432)	
End-end invagination anastomosis	106 (84.1)	194 (67.8)	3.922 (1.143–13.456)		5.510 (1.391–21.821)	
Duct-mucosa anastomosis	11 (8.7)	32 (11.2)	2.396 (0.595–9.642)		7.918 (1.619–38.722)	
Laparoscopic operation				0.002		0.034
Yes	10 (7.9)	3 (1.0)	1		1	
No	116 (92.1)	283 (99.0)	0.128 (0.034–0.477)		0.188 (0.040–0.883)	
Early jejunal nutrition				0.065		
Yes	25 (19.8)	81 (28.3)	1			
No	101 (80.2)	205 (71.7)	1.617 (0.970–2.694)			
Use of somatostatin after PD				0.059		
Yes	84 (66.7)	161 (56.3)	1			
No	42 (33.3)	125 (43.7)	0.655 (0.422–1.017)			
Professional group				0.000		0.000
Yes	38 (30.2)	190 (66.4)	1		1	
No	88 (69.8)	96 (33.6)	4.718 (2.985–7.457)		3.925 (2.250–6.847)	

—: the multivariate analysis of intraoperative blood loss was not performed in this study because of its corresponding relationship with intraoperative blood transfusion. Missing values: 3 diabetes mellitus patients and 2 hypertension patients were missing. OR: odds ratio; CI: confidence interval; OR_adj_: adjusted ORs presented with 95% CI.

**Table:** (a) Patient-related factors for CR-PF

Variants	CR-PF	NOCR-PF	Univariate analysis		Multivariate analysis	
	No. (%)	No. (%)	OR_adj_ (95% CI)	*P*	OR_adj_ (95% CI)	*P*
Smoking				0.84		
Yes	19 (25.7)	83 (24.6)	1			
No	55 (74.3)	255 (75.4)	0.942 (0.529–1.678)			
Heavy smoking (cigarettes/day)				0.191		
≥20	15 (20.3)	48 (14.2)	1			
<20	59 (79.3)	290 (85.8)	0.651 (0.342–1.239)			
Excessive drinking				0.493		
Yes	18 (24.3)	70 (20.7)	1			
No	56 (75.7)	268 (79.3)	0.813 (0.449–1.470)			
Cholangitis				0.002		
Yes	17 (23.0)	33 (9.8)	1			
No	57 (77.0)	305 (90.2)	0.363 (0.189–0.695)			
Cholecystitis				0.075		0.004
Yes	63 (85.1)	255 (75.4)	1		1	
No	11 (14.9)	83 (24.6)	0.536 (0.270–1.066)		0.321 (0.150–0.690)	
Jaundice				0.054		
Yes	45 (60.8)	244 (72.2)	1			
No	29 (39.2)	94 (27.8)	1.673 (0.991–2.825)			
Coronary heart disease				0.284		
Yes	8 (10.8)	24 (7.1)	1			
No	66 (89.2)	314 (92.9)	0.631 (0.271–1.465)			
Hypertension				0.572		
Yes	9 (12.3)	34 (10.1)	1			
No	64 (87.7)	303 (89.9)	0.798 (0.365–1.745)			
Diabetes mellitus				0.163		
Yes	4 (5.5)	37 (11.0)	1			
No	69 (94.5)	299 (89.0)	2.135 (0.736–6.188)			
Preoperative serum total bilirubin (*μ*mol/L)				0.412		
≤17.1	19 (25.7)	72 (21.3)	1			
>17.1	55 (74.3)	266 (78.7)	0.784 (0.437–1.404)			
Preoperative hemoglobin (g/L)				0.324		
<90	3 (4.1)	7 (2.1)	1			
≥90	71 (95.9)	331 (97.9)	0.501 (0.126–1.983)			
Preoperative serum albumin (g/L)				0		0.000
<30	9 (12.2)	7 (2.1)	1		1	
≥30	65 (87.8)	331 (97.9)	0.153 (0.055–0.425)		0.107 (0.031–0.363)	
Postoperative serum albumin (g/L)				0.971		
<30	26 (35.1)	118 (34.9)	1			
≥30	48 (64.9)	220 (65.1)	0.990 (0.585–1.677)			
Primary site of disease				0.935		
Caput pancreatis	34 (45.9)	154 (45.6)	1			
Duodenum	23 (31.1)	100 (29.6)	1.042 (0.580–1.872)			
Biliary ducts	17 (23.0)	84 (24.9)	0.917 (0.483–1.738)			
Pathologic diagnosis				0.322		
Caput pancreatis cancer	28 (37.8)	111 (32.8)	1			
Duodenal cancer	13 (17.6)	68 (20.1)	0.758 (0.368–1.563)			
Cholangiocarcinoma	15 (20.3)	77 (22.8)	0.772 (0.387–1.542)			
Pancreatitis	1 (1.4)	24 (7.1)	0.165 (0.021–1.274)			
Carcinoma of ampulla	4 (5.4)	22 (6.5)	0.721 (0.230–2.261)			
Others	13 (17.6)	36 (10.7)	1.432 (0.671–3.054)			
Texture of the remnant pancreas				0.013		0.044
Hard	9 (12.2)	88 (26.0)	1		1	
Soft	65 (87.8)	250 (74.0)	2.542 (1.215–5.319)		2.316 (1.205–5.234)	
Diameter of pancreatic duct (mm)				0.400		
<3	41 (55.4)	169 (50.0)	1			
≥3	33 (44.6)	169 (50.0)	0.805 (0.485–1.334)			

**Table:** (b) Operative- and therapeutic-related factors for CR-PF

Variants	CR-PF	NOCR-PF	Univariate analysis		Multivariate analysis	
	No. (%)	No. (%)	OR_adj_ (95% CI)	*P*	OR_adj_ (95% CI)	*P*
Preoperative biliary drainage treatment				0.068		
Yes	11 (14.9)	27 (8.0)	1			
No	63 (85.1)	311 (92.0)	0.497 (0.235–1.054)			
Operative time (min)				1.081		
<295	11 (14.9)	68 (20.1)	1			
≥295	63 (85.1)	270 (79.9)	0.693 (0.347–1.387)			
Intraoperative blood loss (mL)				0.004	—	—
<300	20 (27.0)	136 (40.2)	1		—	—
300–600	14 (18.9)	97 (28.7)	0.981 (0.473–2.039)		—	—
600–900	20 (27.0)	53 (15.7)	2.566 (1.279–5.148)		—	—
≥900	20 (27.0)	52 (15.4)	2.615 (1.302–5.253)		—	—
Intraoperative blood transfusion (mL)				0.002		0.001
<300	19 (25.7)	158 (46.7)	1		1	
300–600	18 (24.3)	72 (21.3)	2.079 (1.030–4.196)		2.311 (1.049–5.092)	0.038
600–900	18 (24.3)	70 (20.7)	2.138 (1.058–4.321)		2.657 (1.207–5.851)	0.015
≥900	19 (25.7)	38 (11.2)	4.158 (2.008–8.609)		5.337 (2.301–12.376)	0.000
Pancreatic duct stent drainage				0.007		0.542
No stent	24 (32.4)	65 (19.2)	1		1	
Internal drainage	45 (60.8)	206 (60.9)	0.592 (0.335–1.045)		0.692 (0.359–1.334)	0.272
External drainage	5 (6.8)	67 (19.8)	0.202 (0.073–0.562)		0.814 (0.248–2.671)	0.375
Excision method				0.068		
Without PP	63 (85.1)	311 (92.0)	1			
With PP	11 (14.9)	27 (8.0)	2.011 (0.949–4.264)			
Methods of anastomosis				0.069		
Binding anastomosis	2 (2.7)	23 (6.8)	1			
End-side invagination anastomosis	3 (4.1)	41 (12.1)	0.841 (0.131–5.409)			
End-end invagination anastomosis	63 (85.1)	237 (70.1)	3.057 (0.702–13.314)			
Duct-mucosa anastomosis	6 (8.1)	37 (10.9)	1.865 (0.347–10.034)			
Laparoscopic operation				0.231		
Yes	4 (5.4)	9 (2.7)	1			
No	70 (94.6)	329 (97.3)	0.479 (0.143–1.599)			
Early jejunal nutrition				0.238		
Yes	15 (20.3)	91 (26.9)	1			
No	59 (79.7)	247 (73.1)	1.449 (0.783–2.682)			
Use of somatostatin after PD				0.069		
Yes	51 (68.9)	194 (57.4)	1			
No	23 (31.1)	144 (42.6)	0.608 (0.355–1.040)			
Professional group				0.000		0.000
Yes	16 (21.6)	212 (62.7)	1		1	
No	58 (78.4)	126 (37.3)	6.099 (3.362–11.066)		5.674 (2.867–11.230)	

—: the multivariate analysis of intraoperative blood loss was not performed in this study because of its corresponding relationship with intraoperative blood transfusion. Missing values: 3 diabetes mellitus patients and 2 hypertension patients were missing. OR: odds ratio; CI: confidence interval; OR_adj_: adjusted ORs presented with 95% CI.

**Table 4:** Hospital charges and hospital stays.

	NOPF	PF	NOCR-PF	CR-PF
Average hospital stays (d)				
Normality test *P*	0.009	0.024	0.004	0.06
*t*-test/nonparametric test *P*		0.000^a^		0.000^b^
Mean (d)	22.25	39.08	23.33	46.42
Standard deviation				15.80

Average hospital charges				
Normality test *P*	0.223	0.617	0.279	0.915
*t*-test/nonparametric test *P*		0.000^a^		0.001^b^
Mean (yuan)	56323.47	83347.93	61339.84	81448.18
Standard deviation	24360.81	32007.30	28166.70	31699.74

Missing values: data missing for 8 patients in hospital charges. ^a^ *P* value of nonparametric test between PF and NOPF groups; ^b^ *P* value of *t* test between NOCR-PF and CR-PF groups.
